# Editorial: Case reports in frontiers in gastroenterology

**DOI:** 10.3389/fgstr.2024.1458278

**Published:** 2024-08-06

**Authors:** Michele Di Stefano

**Affiliations:** Internal Medicine Unit, Istituto di Ricovero e Cura a Carattere Scientifico (IRCCS) S. Matteo Hospital Foundation, Pavia, Italy

**Keywords:** case report, small bowel carcinoma, hiatal hernia, colonic stenting, endoscopic procedures

Medical research is becoming increasingly driven towards the explanation of in-depth disease mechanisms, disentangling pathophysiology to trace new therapeutic approaches. The great interest in these study types has gradually led to a radical modification of medical press editorial policy, with many medical journals excluding the publication of case reports. This modification may be justifiable due to the large impact on our knowledge papers describing important advances in understanding on, for instance, the role of the mucosal immune system in the regulation of many inflammatory, endocrine, and metabolic pathways have. However, it is also true that case reports, leaning on unique or rare aspects of patient clinical management, may stimulate the reader’s interest, adding a tile to her/his expertise. They also represent a valuable educational tool, improving both inductive and deductive reasoning and thus improving decision-making. Accordingly, a Research Topic on Case Reports in Gastroenterology including papers dealing with diagnostic and therapeutic approaches of uncommon complications or conditions was proposed.

The paper series includes clinical experiences of patients with neoplastic conditions undergoing unusual complications, suffering from an unknown advanced disease unmasked during the diagnostic approach of an apparently unrelated clinical presentation or suffering from an acute complication of immunotherapy and showing a promising therapeutic response. Moreover, a mini-invasive approach to hiatal hernia, the repair of a rectovaginal fistula with an innovative endoscopic approach, and a respiratory complication during a common operative endoscopic procedure were also reported. These cases are noteworthy for many reasons.

Bressington et al. reported on a patient with colorectal cancer who underwent self-expanding metal stent (SEMS) positioning in a palliative setting. Endoscopic SEMS offers the advantage of avoiding surgical stoma formation but, in a palliative setting, data on the effectiveness of this procedure are available only for a mean one-year follow up (1, 2). However, a mean survival after SEMS positioning of 18 months was recently reported (3) and the descripted case reported on a patient showing the failure of the SEMS after 30 months from its positioning, suggesting the need for data on long-term outcomes.

Mostafa et al. reported the case of a patient with tension pneumothorax caused by a herniation of the transverse colon through the esophageal hiatus after minimally invasive esophagectomy and gastric pull-up associated with intrathoracic colonic obstruction and perforation. Gastric and small bowel herniation after esophagectomy is a common finding, but isolated colonic herniation is rare. This is the distinctive feature of this case. Accordingly, the main point of the paper is the suggestion of the need for a prompt examination of patients developing both abdominal and respiratory symptoms, even a long time after esophagectomy.

Amaris et al. reported the case of a patient who underwent cutaneous malignant melanoma exeresis, presenting to a cardiologist due to angina, associated with asthenia and lightheadedness suspected of a cardiac origin. Anemia on direct oral anticoagulant treatment was present and this condition already explained the symptoms. However, upper and lower endoscopies revealed the presence of a rare, synchronous gastric and colonic metastasis, part of a wide metastatic involvement of brain, lung, and bone. The patient was successfully treated with nivolumab and stereotactic radiosurgery of brain metastasis. A two-year follow up was performed. The peculiarity of this case relies on the need to underline the presence of iron-deficiency anemia in patients with a history of melanoma due to the efficacy of immunotherapy in reverting the poor prognosis (4) of patients with gastrointestinal metastatic involvement.

The case report of Torrado et al. discusses the need for a strict monitoring of splenectomized patients undergoing immunotherapy, besides vaccinations. In the descripted case, CTLA4 and anti-PD1 treatment caused a fatal overwhelming post-splenectomy septicemia with *S. pneumoniae* isolation in blood. The case report underlines both the need to complete the vaccination schedule after splenectomy and the difficulties of management and therapeutic decisions in cancer patients suitable for immunotherapy but affected by comorbidities known to worsen the patient immune response.

The relevance of the case reported by Fu et al. relies on the opportunity to treat small bowel adenocarcinoma with an association between CapeOx regimen and bevacizumab in a palliative setting. The diagnosis of this condition represents a very difficult task due to unspecific clinical presentation, causing an important diagnostic delay and, in turn, an advanced stage diagnosis, increasing the number of patients unsuitable for surgery. The case reported the stabilization of clinical conditions in a patient with a stage IV small bowel adenocarcinoma, during a follow up longer than the reported average for the disease stage (5).

Three case reports dealt with benign disorders. Turaga et al. described a combined endoscopic and surgical approach for a large hiatal hernia repair involving the stomach and bowel. An open approach allowed the reduction of the large hernia and transoral incisionless fundoplication completed the procedure. The case suggests how combined approaches may be necessary, particularly when adhesions limit mini-invasive procedure safety, but also confirms non-surgical procedures may be used alongside surgery to treat complex conditions.

He et al. described a successful combined transanal and transvaginal endoscopic approach to repair a rectovaginal fistula secondary to unsuccessful hemorrhoid surgery. The procedure is based on the creation of a mucosal pinhole, followed by electrocauterization by argon plasma coagulator and, finally, a purse-string suture at both the rectal and vaginal side. This new technique should be taken into consideration for the treatment of large rectovaginal fistulas characterized by a high risk of recurrence (6).

Siblani et al. described a complication of endoscopic gastrostomy feeding tube removal during a programmed endoscopic procedure. Percutaneous endoscopic gastrostomy is commonly used to guarantee an adequate caloric support in many patients unable to meet caloric allowance through oral intake. However, complications during both positioning and removal are described (7, 8). In this case report, during the tube removal, at the proximal esophagus level a rotation of the tube caused its blockage and a perforation in the pharynx required surgery. Suggested strategies to minimize the risk of complication of PEG removal were the use of a cover tube and the tube being at least 3 cm away from the bumper. Endoscopists should also pay close attention to patients with difficult anatomy to reduce the risk of procedural complications.

I hope this Research Topic will catch the interest of many readers and will induce a critical consideration and revision of personal experiences to gain some new information and improve our clinical activity.

## Author contributions

MDS: Conceptualization, Data curation, Formal analysis, Investigation, Methodology, Project administration, Software, Supervision, Validation, Visualization, Writing – original draft, Writing – review & editing.
